# Molecular identification of non-tuberculous mycobacteria isolated from clinical specimens in Zambia

**DOI:** 10.1186/s12941-014-0059-8

**Published:** 2015-01-16

**Authors:** Grace Mwikuma, Geoffry Kwenda, Bernard M Hang’ombe, Edgar Simulundu, Trevor Kaile, Selestine Nzala, Seter Siziya, Yasuhiko Suzuki

**Affiliations:** School of Medicine, University of Zambia, Lusaka, Zambia; School of Veterinary Medicine, University of Zambia, Lusaka, Zambia; School of Medicine, Copperbelt University, Kitwe, Zambia; Hokkaido University Research Center for Zoonosis Control, Sapporo, Japan

**Keywords:** Non-tuberculous mycobacteria, Identification, Zambia

## Abstract

**Background:**

The emergence of Acquired Immunodeficiency Syndrome has highlighted the increased incidence and importance of the disease caused by Non-tuberculous Mycobacteria (NTM). While disease due to *M. avium*-*intracellulare* complex is apparently common throughout the world, other Non-tuberculous mycobacterial species have been isolated from both immunocompromised and immunocompetent individuals. The increasing number of infections caused by these organisms has made it clinically important to quickly identify mycobacterial species. The diagnosis of a pathogenic versus a non-pathogenic species not only has epidemiological implications but is also relevant to the demands of patient management. Since antibiotic treatment varies according to the species encountered, species identification would reduce the burden of some of these emerging opportunistic pathogens especially in immunocompromised patients and improve their quality of life.

**Findings:**

A total of 91 NTM suspected isolates from four regions of Zambia were included in the study. These isolates were identified using the sequence analysis of the *16S-23S* rRNA intergenic transcribed spacer (ITS) region of Mycobacteria.

Fifty-four of the 91 (59%) isolates were identified as NTM and these included *M. intracellulare* (27.8%), *M. lentiflavum* (16.7%), *M. avium* (14.8%), *M. fortuitum* (7.4%), *M. gordonae* (7.4%), *M. kumamotonense* (3.7%), *M. indicus pranii* (3.7%), *M. peregrinum* (3.7%), *M. elephantis* (1.85%), *M. flavescens* (1.85%), *M. asiaticum* (1.85%), *M. bouchedurhonense* (1.85%), *M. chimaera* (1.85%), *M. europaeum* (1.85%), *M. neourum* (1.85%), *M. nonchromogenicum* (1.5%).

**Conclusion:**

The study has shown that DNA sequencing of the ITS region may be useful in the preliminary identification of NTM species. All species identified in this study were potentially pathogenic.

**Electronic supplementary material:**

The online version of this article (doi:10.1186/s12941-014-0059-8) contains supplementary material, which is available to authorized users.

## Findings

Members of the genus *Mycobacterium* are important causes of respiratory disease, thereby posing an important public health threat to people and animals worldwide. Recently, there has been increased cognisance of a variety of diseases that have been caused by Non-tuberculous Mycobacteria (NTM) [1]. The current unprecedented high level of interest in NTM infections is mainly the result of the association of NTM infection with immune-suppression [2] and the recognition that NTM pulmonary infections are encountered with increasing frequency in the immune-competent patients. Another major factor contributing to the increased awareness of the importance of NTM as human pathogens is the improvement in the mycobacteriology laboratory techniques, resulting in enhanced isolation and more rapid and accurate identification of NTM from clinical specimens [3]. Consistent with advances in mycobacteriological laboratory techniques is the emphasis on the identification of individual NTM species and the clinical disease-specific syndromes they produce [4]. The number of NTM species has been steadily increasing [5] and currently there are more than 160 NTM species [6].

Although the reservoir of infection in most cases remains unclear, there is a general notion that NTM infections are derived mainly from the environment. NTM are widely distributed in nature and have been isolated from water and soil with water being the major reservoir [7]. There are a variety of situations where human and mycobacterial geographical and environmental distributions can overlap leading to exposure of humans. A major overlap occurs with water where humans are exposed to mycobacteria in water through drinking, swimming and bathing [8]. Aerosols generated during some of these activities can also lead to human exposure [9]. The presence of NTM in water, coupled with their disinfectant resistance, leads to their presence in hot tubs, solutions used in medical treatments and water–oil emulsions used to cool metal working tools [10]. It is however, generally believed that the majority of human-mycobacterial interactions are transient, self-curing colonisations [11,12]. These subclinical human-mycobacterial interactions may give a transient stimulation of certain pathways that may set the stage for manifestation of other diseases [4].

Non-tuberculous Mycobacteria are often involved in nosocomial outbreaks [13], although there is little or no evidence for person-to-person transmission of these organisms [3]. However, the significance of isolation of these organisms in clinical samples remains unclear since the number of diseases they cause is difficult to assess and no system for notification exists as in the case of *M. tuberculosis*. In addition, treatment and infection control measures vary according to the aetiological species [3]. Therefore, rapid and accurate identification of mycobacteria to the species level is essential to facilitate early treatment of mycobacterioses.

Zambia is a high burden country for tuberculosis and patients with chronic pneumonia, lymphadenitis, pyrexia of unknown origin and other chronic infections are evaluated for tuberculosis through microbiological cultures of various clinical specimens. In the process of isolating *M. tuberculosis*, NTM are also isolated from these specimens, without any attempt to identify them to species level. Therefore this study was initiated to identify NTM to species level for ease of managing such suspect conditions.

## Materials and methods

This was a retrospective study of 91 isolates stored over a period of three and half years from January 2009 to June 2012 from four regions of Zambia (Eastern, Lusaka, Southern and Western). The stored isolates were revived using Lowestein Jensen (LJ) and Mycobacterium Growth Indicator Tube (MGIT) by standard microbiological procedures [14]. The cultures were then subjected to PCR identification and DNA sequencing of the 23S rRNA (ITS) region with primers Sp1 (5′-ACC TCC TTT CTA AGG AGC ACC-3′) and Sp2 (5′-GAT GCT CGC AAC CAC TAT CCA-3′) [15]. The obtained sequences were compared with those available in GenBank by BLAST searches. Sequences that displayed at least 98% sequence identity when compared to those in the GenBank were preliminary considered as identified species [16].

## Results

**Figure 1:**
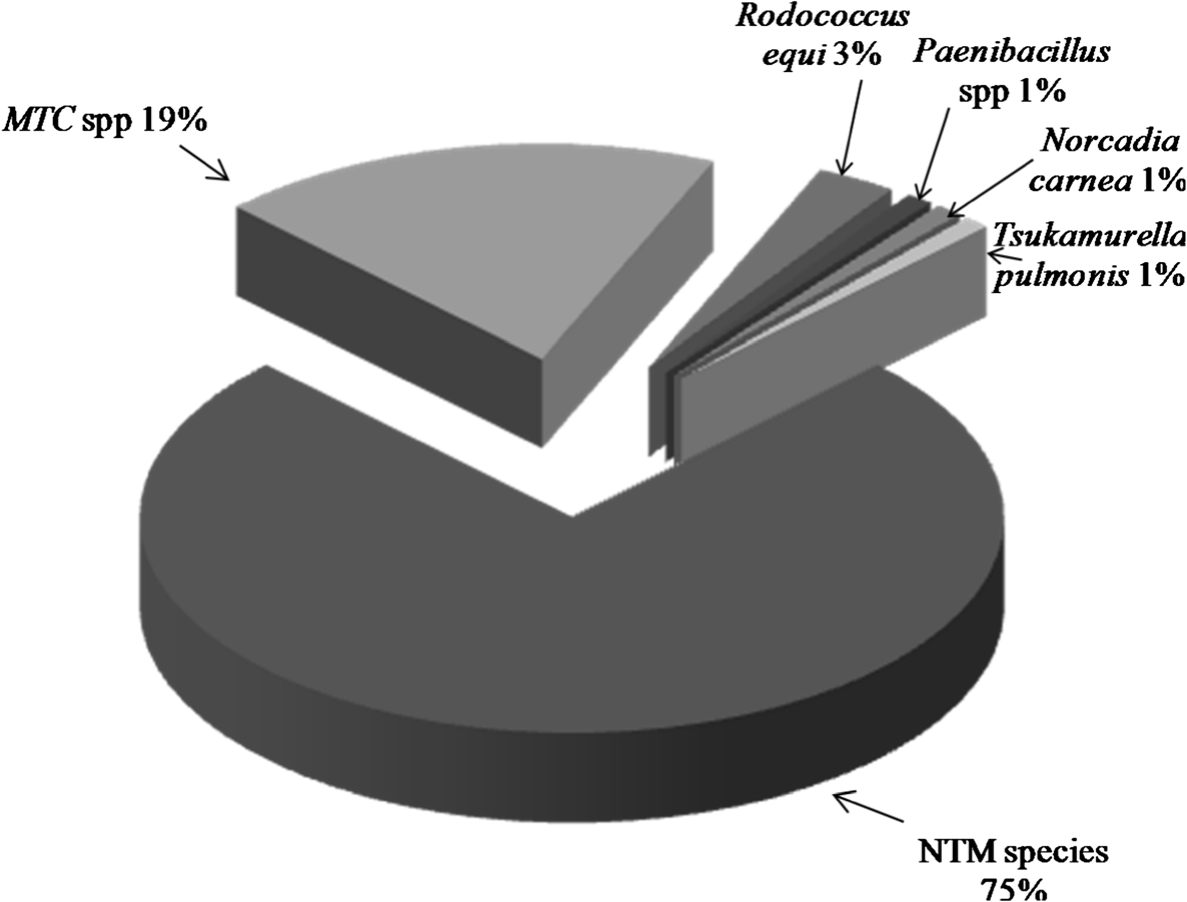
**Organisms identified by sequence analysis of the ITS positive PCR amplicons.**

**Figure 2:**
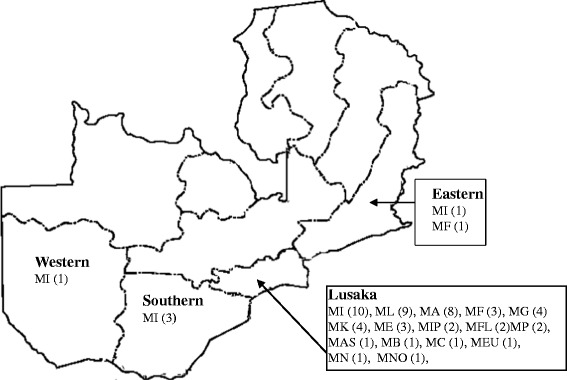
**Map of Zambia showing regions of various NTM identified.** The regions are indicated in bold with the identified NTM. MI (*M. intracellulare*), ML (*M. lentiflavum*), MA (*M. avium*), MF (*M. fortuitum*), MG (*M. gordonae*), MK (*M. kumamotonense*), ME (*M. elephantis*), MIP (*M. indicus pranii*), MFL (*M. flavescens*), MP (*M. peregrinum*), MAS (*M. asiaticum*), MB (*M. bouchedurhonense*), MC (*M. chimaera*), MEU (*M. europaeum*), MN (*M. neoaurum*), MNO (*M. nonchromogenicum*).

Sequence analysis and identification of the ITS region of the 91 strains showed: NTM species (68), *Mycobacterium tuberculosis* complex (17), *Rodococcus equi* (3), *Tsukamurella pulmonis* (1), *Norcadia carnea* (1) and *Paenibacillus* species (1) as shown in Figure 1 and Additional file 1: Table S1. Of the 68 NTM isolates, 54 were identified to species level as shown in Table 1, while 14 could not be identified. The 54 NTM species identified belonged to 16 different species with *M. intracellulare* exhibiting the highest frequency of identity (Additional file 2). Furthermore, *M. intracellulare* was the only NTM specie identified in the four regions of Zambia under study, with Lusaka region having a higher frequency (10), Southern (3), Western (1) and Eastern (1). *M. fortuitum* was identified in the Eastern and Lusaka regions, with one and three isolates respectively. All the other 14 species identified were from the region of Lusaka. A map of Zambia showing regions of distribution of various NTM identified in this study is shown in Figure 2.

**Table 1:** **Spectrum and Identity of NTM species**

**NTM species**	**No.**	**Frequency (%)**
*M. intracellulare*	15	27.8
*M. lentiflavum*	9	16.7
*M. avium*	8	14.8
*M. fortuitum*	4	7.41
*M. gordonae*	4	7.41
*M. kumamotonense*	2	3.70
*M. indicus pranii*	2	3.70
*M. peregrinum*	2	3.70
*M. elephantis*	1	1.85
*M. flavescens*	1	1.85
*M. asiaticum*	1	1.85
*M. bouchedurhonense*	1	1.85
*M. chimaera*	1	1.85
*M. europaeum*	1	1.85
*M. neoaurum*	1	1.85
*M. nonchromogenicum*	1	1.85
**Total**	**54**	**100**

## Discussion

Non-tuberculous Mycobacteria have gained a lot of clinical significance in the last couple of decades in immunocompromised and immunocompetent individuals or patients [2]. Their ubiquitous distribution in nature put them at an advantage of having hosts close to ecological niches compounded by human activities. This might be the first study in Zambia to identify NTM species using PCR and DNA sequencing of the ITS region. This study has provided a range of NTM species which are potentially pathogenic. A total of 64 isolates were initially identified as NTM species. On sequencing and GenBank comparison, only 54 were identified to species level using the preliminary identification strategy which has been previously described [16]. The most prevalent species was *M. intracellulare* followed by *M. lentiflavum* and *M. avium*. This was in partial agreement with the findings of the study conducted by Buijtels and others [17] in the Eastern region of Zambia where sputum Mycobacterial culture isolates were identified by 16S rRNA gene sequencing. In this study *M. fortuitum*, was isolated from a clinical case. The other studies conducted in the Western and Northern regions of Zambia [18] and other parts of the world [19,20] were in contrast with these findings. The reason for this difference is that NTM species distribution differs from one geographical region to another [21].

*M. intracellulare* has been identified as the important species of the *Mycobacterium avium* complex. It has been identified together with *M. avium* as a complex because of their close similarities. *M. intracellulare* has been found to be more pathogenic than *M. avium* [22] and have been reported to cause disease not only in immunocompromised but also in immunocompetent subjects [23]. Other NTM species such as *M. lentiflavum* and *M. avium* have been implicated in clinical disease of immunocompromised as well as immunocompetent individuals [24,25]. *M. lentiflavum* has been isolated from various human specimens including pleural effusions, ascites and lung tissue [26,27] and have mainly been associated with causing an array of infections in immunocompromised patients [28]. Unlike *M. intracellulare,* most *M. avium* species do not multiply in monocytes of healthy individuals [29]. *M. fortuitum* infrequently cause a variety of diseases including bone and soft tissue infections, lymphadenitis and post-surgical infections and lung disease [30]. *M. kumamotonense, M. indicus pranii, M. flavescens, M. bouchedurhonense, M. chimaera, M. europaeum* and *M. nonchromogenicum* were identified and reported for the first time in Zambia. Some of these NTM have been associated with clinical disease [31,32] while *M. indicus pranii* is an atypical saprophytic bacterium that has raised a lot of research interest in leprosy immunotherapeutic [33]. *M. flavescens* has been isolated from the synovial fluid of an AIDS patient [34], whereas *M. bouchedurhonense* and *M. chimaera* have been documented in some respiratory tract infections [35]. *M. europaeum* was isolated from the sputum samples of an Iranian human immunodeficiency virus-infected patient and a cystic fibrosis patient with chronic pulmonary disease [36] while *M. nonchromogenicum* has been associated with sarcoidosis [37].

Other organisms which are not NTM that were identified include *Mycobacterium tuberculosis* complex species, *Rodococcus equi, Nocardia carnea, Tsukamura pulmonis* and *Paenibacillus* species. Of significance is the identification of *Rodococcus equi* from a clinical specimen in Zambia. This is the second time *Rodococcus equi* has been reported in Zambia [38]. The organisms: *Rodococcus equi, Nocardia carnea, Tsukamura pulmonis* and *Paenibacillus* species have been known to cause pulmonary diseases that are similar to tuberculosis [39-41]. Management of infections by these agents is different from that of tuberculosis. Therefore species identification of NTM remains of great importance as it provides an opportunity to develop a database that may help increase the scope of mycobacterioses.

## Availability of supporting data

The data supporting the results of this study are included within this article.

## Additional files

Additional file 1: Table S1. Species with the highest degree of nucleotide sequence identity to isolates from Zambia. Additional file 2:
**Nucleotide sequences of Zambian isolates.**

## Abbreviations

NTM: Non-tuberculous mycobacteria
MGIT: Mycobacteria growth indicator tube
PCR: Polymerase chain reaction
LJ: Lowestein Jensen
ITS: Intergenic transcribed spacer
