# Supplementary material for: Molecular identification of non-tuberculous mycobacteria isolated from clinical specimens in Zambia
Source: Ann Clin Microbiol Antimicrob. 2015 Jan 16;14:1. doi: 10.1186/s12941-014-0059-8 (PMC4302154; doi:10.1186/s12941-014-0059-8)
Supplement: Additional file 1: Table S1. — Species with the highest degree of nucleotide sequence identity to isolates from Zambia. [file 12941_2014_59_MOESM1_ESM.docx]

| **Additional file 1: Table S1.** Species with the highest degree of nucleotide sequence identity to isolates from Zambia | | | | | | |
| --- | --- | --- | --- | --- | --- | --- |
| **Serial No.** | **Isolate No.** | **Species with highest degree of sequence identity** | **Identity (%)** | **E Value** | **GenBank Accession No.** |  |
| 1 | N001R | *M. tuberculosis strain ZMC13-88* | 100 | 3e-148 | CP009101 |  |
| 2 | N002R | *M. intracellulare* | 99 | 0.0 | [KF186681](http://www.ncbi.nlm.nih.gov/nucleotide/534471402?report=genbank&log$=nuclalign&blast_rank=1&RID=9JUK74VA015) |  |
| 3 | N003R | *Tsukamurella pulmonis* BC5473 | 88 | 0.0 | EU086531 |  |
| 4 | N004R | *M. fortuitum* | 96 | 6e-67 | KF366427 |  |
| 5 | N005R | *M. fortuitum* | 100 | 8e-107 | AM902929 |  |
| 6 | N006R | *M. gordonae* | 100 | 0.0 | [EU497913](http://www.ncbi.nlm.nih.gov/nucleotide/169641044?report=genbank&log$=nuclalign&blast_rank=1&RID=9JTN8WT9014) |  |
| 7 | N007R | *M. intracellulare* | 99 | 0.0 | [KF186681](http://www.ncbi.nlm.nih.gov/nucleotide/534471402?report=genbank&log$=nuclalign&blast_rank=1&RID=9JVDS44A014) |  |
| 8 | N008R | *M. gordonae* | 100 | 0.0 | [EU497913](http://www.ncbi.nlm.nih.gov/nucleotide/169641044?report=genbank&log$=nuclalign&blast_rank=1&RID=9JX8RY2B015) |  |
| 9 | N009R | *M. gordonae* | 92 | 5e-145 | EU497913 |  |
| 10 | N010R | *Paenibacillus sp*. Y412MC10 | 86 | 1e-87 | CP001793 |  |
| 11 | N011R | *M. tuberculosis str. Haarlem* | 99 | 9e-158 | CP001664 |  |
| 12 | N012R | *M. tuberculosis str. Haarlem* | 98 | 0.0 | CP001664 |  |
| 13 | N014R | *M. flavescens* | 88 | 2e-84 | [AF191086](http://www.ncbi.nlm.nih.gov/nucleotide/8895226?report=genbank&log$=nuclalign&blast_rank=1&RID=9T1TVW1001R) |  |
| 14 | N015R | *M. flavescens* | 88 | 6e-30 | [AF191086](http://www.ncbi.nlm.nih.gov/nucleotide/8895226?report=genbank&log$=nuclalign&blast_rank=1&RID=9T29TMY501R) |  |
| 15 | N016R | *M. tuberculosis strain ZMC13-88* | 100 | 3e-148 | CP009101 |  |
| 16 | N020R | *M. avium* | 100 | 0.0 | [FJ858760](http://www.ncbi.nlm.nih.gov/nucleotide/289718555?report=genbank&log$=nuclalign&blast_rank=1&RID=B6U78F3H014) |  |
| 17 | N021R | *M. tuberculosis isolate 1371* | 100 | 4e-110 | DQ131568 |  |
| 18 | N023R | *M. timonense* | 95 | 4e-84 | [EF591050](http://www.ncbi.nlm.nih.gov/nucleotide/157503928?report=genbank&log$=nuclalign&blast_rank=1&RID=9KCC0BUH014) |  |
| 19 | N027R | *M. gordonae* | 99 | 0.0 | [FJ858761](http://www.ncbi.nlm.nih.gov/nucleotide/289718556?report=genbank&log$=nuclalign&blast_rank=1&RID=9KN0VHYP015) |  |
| 20 | N031R | *M. europaeum* | 99 | 2e-180 | [HM022198](http://www.ncbi.nlm.nih.gov/nucleotide/295815604?report=genbank&log$=nuclalign&blast_rank=1&RID=9KNUMP48014) |  |
| 21 | N033R | *M. intracellulare* | 99 | 0.0 | [HM454238](http://www.ncbi.nlm.nih.gov/nucleotide/320171719?report=genbank&log$=nuclalign&blast_rank=1&RID=BE5GNPJ0014) |  |
| 22 | N038R | *M. tuberculosis str. Haarlem* | 100 | 0.0 | CP001664 |  |
| 23 | N039R | *M. senegalense* | 96 | 3e-137 | FJ858767 |  |
| 24 | N041R | *M. tuberculosis H37Rv* | 100 | 3e-131 | CP003248 |  |
| 25 | N044R | *M. tuberculosis* RGTB327 | 100 | 2e-108 | CP003233 |  |
| 26 | N047R | *Rhodococcus equi* 103S | 99 | 0.0 | FN563149 |  |
| 27 | N049R | *M. avium* | 98 | 0.0 | [FJ858758](http://www.ncbi.nlm.nih.gov/nucleotide/289718553?report=genbank&log$=nuclalign&blast_rank=1&RID=9KS9GGJ9014) |  |
| 28 | N050R | *M. tuberculosis EA15* | 100 | 0.0 | CP006578 |  |
| 29 | N051R | *M. arupense* | 86 | 3e-138 | [DQ168663](http://www.ncbi.nlm.nih.gov/nucleotide/75993575?report=genbank&log$=nuclalign&blast_rank=1&RID=BBB82G4F014) |  |
| 30 | N052R | *M. tuberculosis H37Rv* | 80 | 2e-64 | CP003248 |  |
| 31 | N054R | *M. tuberculosis strain ZMC13-88* | 100 | 3e-148 | CP009101 |  |
| 32 | N055R | *M. indicus pranii* | 100 | 0.0 | [CP002275](http://www.ncbi.nlm.nih.gov/nucleotide/405126727?report=genbank&log$=nuclalign&blast_rank=1&RID=9NMJVMFB014) |  |
| 33 | N062R | *M. intracellulare* | 99 | 0.0 | [HM454237](http://www.ncbi.nlm.nih.gov/nucleotide/320171718?report=genbank&log$=nuclalign&blast_rank=1&RID=9NUFSPMD014) |  |
| 34 | N064R | *M. bouchedurhonense* | 99 | 4e-156 | EF591051 |  |
| 35 | N065R | *M. intracellulare* | 99 | 0.0 | [KF186681](http://www.ncbi.nlm.nih.gov/nucleotide/534471402?report=genbank&log$=nuclalign&blast_rank=1&RID=9NVARC40014) |  |
| 36 | N066R | *M. tuberculosis H37Rv* | 99 | 4e-127 | CP003248 |  |
| 37 | N068R | *M. tuberculosis 7199-99* | 98 | 2e-174 | HE663067 |  |
| 38 | N069R | *M. intracellulare* | 99 | 0.0 | [HM454238](http://www.ncbi.nlm.nih.gov/nucleotide/320171719?report=genbank&log$=nuclalign&blast_rank=1&RID=BBBA462N014) |  |
| 39 | N070R | *M. tuberculosis str. Haarlem* | 100 | 0.0 | CP003248 |  |
| 40 | N074R | *M. intracellulare* | 99 | 0.0 | [M454237](http://www.ncbi.nlm.nih.gov/nucleotide/320171718?report=genbank&log$=nuclalign&blast_rank=1&RID=9NWAH5DR014) |  |
| 41 | N075R | *M. intracellulare* | 99 | 0.0 | [HM454237](http://www.ncbi.nlm.nih.gov/nucleotide/320171718?report=genbank&log$=nuclalign&blast_rank=1&RID=9NX4DRAS015) |  |
| 42 | N076R | *M. fortuitum* | 100 | 0.0 | [AJ291588](http://www.ncbi.nlm.nih.gov/nucleotide/11322475?report=genbank&log$=nuclalign&blast_rank=1&RID=9NYMZ9NJ014) |  |
| 43 | N077R | *M. intracellulare* | 100 | 8e-153 | [HM454238](http://www.ncbi.nlm.nih.gov/nucleotide/320171719?report=genbank&log$=nuclalign&blast_rank=1&RID=9P1M14JX015) |  |
| 44 | N078R | *Rhodococcus equi* 103S | 99 | 0.0 | FN563149 |  |
| 45 | N083R | *Rhodococcus equi* | 100 | 7e-164 | AF536491 |  |
| 46 | N085R | *M. intracellulare* | 99 | 9e-127 | [KF186681](http://www.ncbi.nlm.nih.gov/nucleotide/534471402?report=genbank&log$=nuclalign&blast_rank=1&RID=9RN8A039014) |  |
| 47 | N089R | *M. intracellulare* | 99 | 0.0 | [HM454237](http://www.ncbi.nlm.nih.gov/nucleotide/320171718?report=genbank&log$=nuclalign&blast_rank=1&RID=9RPB942P015) |  |
| 48 | N096R | *M. fortuitum* | 99 | 7e-71 | [AM902935](http://www.ncbi.nlm.nih.gov/nucleotide/159147497?report=genbank&log$=nuclalign&blast_rank=1&RID=9RVZSK1S014) |  |
| 49 | N097R | *M. kumamotonense* | 99 | 2e-90 | [FN597647](http://www.ncbi.nlm.nih.gov/nucleotide/313209094?report=genbank&log$=nuclalign&blast_rank=1&RID=BBUFRPCE014) |  |
| 50 | N101R | *M. arupense* | 92 | 2e-93 | [DQ168663](http://www.ncbi.nlm.nih.gov/nucleotide/75993575?report=genbank&log$=nuclalign&blast_rank=1&RID=BBXVX19J014) |  |
| 51 | N102R | *M. elephantis* | 100 | 1e-84 | [HM229790](http://www.ncbi.nlm.nih.gov/nucleotide/299013521?report=genbank&log$=nuclalign&blast_rank=1&RID=BBUGKU9C014) |  |
| 52 | N104R | *M. fortuitum* | 93 | 1e-100 | [KF366434](http://www.ncbi.nlm.nih.gov/nucleotide/532809741?report=genbank&log$=nuclalign&blast_rank=1&RID=BBUHZPZR014) |  |
| 53 | N115R | *M. fortuitum* | 93 | 9e-138 | [AM709726](http://www.ncbi.nlm.nih.gov/nucleotide/146218733?report=genbank&log$=nuclalign&blast_rank=1&RID=BBUJ5YK3014) |  |
| 54 | N116R | *M. kumamotonense* | 99 | 3e-157 | [FN597647](http://www.ncbi.nlm.nih.gov/nucleotide/313209094?report=genbank&log$=nuclalign&blast_rank=1&RID=BE9CHC8N014) |  |
| 55 | N122T | *M. lentiflavum* | 100 | 5e-150 | AF318174 |  |
| 56 | N124T | *M. kumamotonense* | 90 | 1e-71 | [FN597647](http://www.ncbi.nlm.nih.gov/nucleotide/313209094?report=genbank&log$=nuclalign&blast_rank=1&RID=BBUK7A3B014) |  |
| 57 | N128T | *M. avium* | 99 | 0.0 | [FJ858758](http://www.ncbi.nlm.nih.gov/nucleotide/289718553?report=genbank&log$=nuclalign&blast_rank=1&RID=BBUKUXF3014) |  |
| 58 | N129T | *M. intracellulare* | 99 | 0.0 | [HM454238](http://www.ncbi.nlm.nih.gov/nucleotide/320171719?report=genbank&log$=nuclalign&blast_rank=1&RID=BBUMPVBM015) |  |
| 59 | N130T | *M. lentiflavum* | 100 | 1e-151 | AF318174 |  |
| 60 | N131T | *M. lentiflavum* | 100 | 1e-85 | [AF318174](http://www.ncbi.nlm.nih.gov/nucleotide/14091011?report=genbank&log$=nuclalign&blast_rank=1&RID=BC6TWN1P014) |  |
| 61 | N132T | *M. lentiflavum* | 100 | 1e-151 | [AF318174](http://www.ncbi.nlm.nih.gov/nucleotide/14091011?report=genbank&log$=nuclalign&blast_rank=1&RID=BC48N41P014) |  |
| 62 | N133T | *M. lentiflavum* | 100 | 4e-156 | [AF318174](http://www.ncbi.nlm.nih.gov/nucleotide/14091011?report=genbank&log$=nuclalign&blast_rank=1&RID=BC491JRS014) |  |
| 63 | N136T | *M. lentiflavum* | 100 | 0.0 | [AF318174](http://www.ncbi.nlm.nih.gov/nucleotide/14091011?report=genbank&log$=nuclalign&blast_rank=1&RID=9RXV28TM014) |  |
| 64 | N137T | *M. lentiflavum* | 100 | 2e-173 | [AF318174](http://www.ncbi.nlm.nih.gov/nucleotide/14091011?report=genbank&log$=nuclalign&blast_rank=1&RID=9RYS749S015) |  |
| 65 | N141T | *M. asiaticum* | 99 | 4e-171 | [AY722098](http://www.ncbi.nlm.nih.gov/nucleotide/57638871?report=genbank&log$=nuclalign&blast_rank=1&RID=BC49CG31014) |  |
| 66 | N143T | *M. tuberculosis H37Rv* | 100 | 0.0 | CP001664 |  |
| 67 | N155T | *M. intracellulare* | 99 | 0.0 | [HM454238](http://www.ncbi.nlm.nih.gov/nucleotide/320171719?report=genbank&log$=nuclalign&blast_rank=1&RID=BC4A20JX014) |  |
| 68 | N158T | *M. indicus pranii* | 100 | 0.0 | CP002275 |  |
| 69 | N160T | *M. avium* | 99 | 0.0 | FJ858758 |  |
| 70 | N161T | *M. avium* | 99 | 0.0 | [FJ858758](http://www.ncbi.nlm.nih.gov/nucleotide/289718553?report=genbank&log$=nuclalign&blast_rank=1&RID=9S869CEV014) |  |
| 71 | N162T | *M. tuberculosis str. Haarlem* | 100 | 0.0 | CP001664 |  |
| 72 | N163T | *M. lentiflavum* | 99 | 5e-155 | [AF318174](http://www.ncbi.nlm.nih.gov/nucleotide/14091011?report=genbank&log$=nuclalign&blast_rank=1&RID=BDKV9GNP014) |  |
| 73 | N167T | *M. avium* | 97 | 6e-175 | [FJ858758](http://www.ncbi.nlm.nih.gov/nucleotide/289718553?report=genbank&log$=nuclalign&blast_rank=1&RID=BDHFCBSU014) |  |
| 74 | N170T | *M. avium* | 99 | 0.0 | [FJ858758](http://www.ncbi.nlm.nih.gov/nucleotide/289718553?report=genbank&log$=nuclalign&blast_rank=1&RID=9SA2KRES01R) |  |
| 75 | N172T | *M. marinum* | 90 | 6e-135 | [FJ868215](http://www.ncbi.nlm.nih.gov/nucleotide/226934928?report=genbank&log$=nuclalign&blast_rank=1&RID=BDHFXKR7014) |  |
| 76 | N178D | *M. avium* | 98 | 3e-122 | [L07852](http://www.ncbi.nlm.nih.gov/nucleotide/31442879?report=genbank&log$=nuclalign&blast_rank=1&RID=9SMPNHZF014) |  |
| 77 | N187D | *M. tuberculosis str. Haarlem* | 100 | 0.0 | CP001664 |  |
| 78 | N197C | *M. lentiflavum* | 100 | 6e-154 | [AF318174](http://www.ncbi.nlm.nih.gov/nucleotide/14091011?report=genbank&log$=nuclalign&blast_rank=1&RID=BDHMA1VP014) |  |
| 79 | N199C | *M. rhodesiae NBB3* | 86 | 8e-62 | CP003169 |  |
| 80 | N201C | *M. nonchromogenicum* | 100 | 2e-87 | [HM584724](http://www.ncbi.nlm.nih.gov/nucleotide/310752639?report=genbank&log$=nuclalign&blast_rank=1&RID=BDHKV0W6015) |  |
| 81 | N202C | *M. palustre* | 94 | 1e-151 | [HM454240](http://www.ncbi.nlm.nih.gov/nucleotide/320171721?report=genbank&log$=nuclalign&blast_rank=1&RID=BDHPF43J014) |  |
| 82 | N204C | *M. neoaurum* | 98 | 3e-75 | HM584726 |  |
| 83 | N205C | *M. kumamotonense* | 88 | 2e-78 | [FN597646](http://www.ncbi.nlm.nih.gov/nucleotide/313209091?report=genbank&log$=nuclalign&blast_rank=1&RID=BDHR5YKG014) |  |
| 84 | N206C | *M. chimaera* | 99 | 4e-146 | AJ548480 |  |
| 85 | N210C | *M. elephantis* | 90 | 4e-54 | HM229790 |  |
| 86 | N211C | *M. elephantis* | 90 | 4e-54 | HM229790 |  |
| 87 | N218D | *M. lentiflavum* | 100 | 6e-180 | AF318174 |  |
| 88 | N219T | *M. intracellulare* | 99 | 0.0 | [HM454238](http://www.ncbi.nlm.nih.gov/nucleotide/320171719?report=genbank&log$=nuclalign&blast_rank=1&RID=BDHTWWM0014) |  |
| 89 | N222T | *Nocardia carnea* strain ATCC 6847 clone 6 | 88 | 1e-137 | GU236354 |  |
| 90 | N224T | *M. peregrinum* | 99 | 4e-130 | AM396481 |  |
| 91 | N225T | *M. peregrinum* | 98 | 4e-130 | AM396481 |  |
