# Supplementary material for: Molecular identification of non-tuberculous mycobacteria isolated from clinical specimens in Zambia
Source: Ann Clin Microbiol Antimicrob. 2015 Jan 16;14:1. doi: 10.1186/s12941-014-0059-8 (PMC4302154; doi:10.1186/s12941-014-0059-8)
Supplement: Additional file 2: — Nucleotide sequences of Zambian isolates. [file 12941_2014_59_MOESM2_ESM.doc]

>N002R

GATTGGGACGAAGTCGTAACAAGGTAGCCGTACCGGAAGGTGCGGCTGGATCACCTCCTTTCTAAGGAGCACCACGAAAAGCACTCCAATTGGTGGGGTGCGAGCCGTGAGGGGTTCCCGTCTGTAGTGGACGGGGGCCGGGTGCACAACAGCAAATGATTGCCAGACACACTATTGGGCCCTGAGACAACACTCGGTCGATCCGTGTGGAGTCCCTCCATCTTGGTGGTGGGGTGTGGTGTTTGAGTATTGGATAGTGGTTGCGAGCATCTAGATGAGCGCATAGTCCTTGTGGCTGATGCGCTCGTCGAAATGTGTAATTTCTTCTTTGGTTTTTGTGTGTAAGTAAGTGTTTAAGGGGGCATCGTGGATGCCTTGGCATCGAGAGCCGATGAAGGACATGGGAGGCT

>N006R

GATTGGGACGAAGTCGTAACAAGGTAGCCGTACCGGAAGGTGCGGCTGGATCACCTCCTTTCTAAGGAGCACCACGAAGAGCACTCCAATTGGTGGGGTGCGAGCCGTGAGGGGTCATCGTCTGTAGTGGACGAAGACCGGGTGCACGACAACAAGCAAAGCCAGACACACTATTGGGTCCTGAGGCAACACCCTCGGGTGCTGTCCCCCCATCTTGGTGGTGGGGTGTGGTGTTTGAGAATTGGATAGTGGTTGCGAGCATCAAAATGTATGCGTTGTCGTCCGCGGCAACGTGTTCTTTTTGTGCAATTTTATTCTTTGGTTTTTGTAGTGTTTGTAAGTGTCTAAGGGCGCATGGTGGATGCCTTGGCATCGAGAGCCGATGAAGGACGTGGGAGGCT

>N007R

GATTGGGACGAAGTCGTAACAAGGTAGCCGTACCGGAAGGTGCGGCTGGATCACCTCCTTTCTAAGGAGCACCACGAAAAGCACTCCAATTGGTGGGGTGCGAGCCGTGAGGGGTTCCCGTCTGTAGTGGACGGGGGCCGGGTGCACAACAGCAAATGATTGCCAGACACACTATTGGGCCCTGAGACAACACTCGGTCGATCCGTGTGGAGTCCCTCCATCTTGGTGGTGGGGTGTGGTGTTTGAGTATTGGATAGTGGTTGCGAGCATCTAGATGAGCGCATAGTCCTTGTGGCTGATGCGCTCGTCGAAATGTGTAATTTCTTCTTTGGTTTTTGTGTGTAAGTAAGTGTTTAAGGGCGCATGGTGGATGCCTTGGCATCGAGAGCCGATGAAGGACGTGGGAGGCT

>N008R

GATTGGGACGAAGTCGTAACAAGGTAGCCGTACCGGAAGGTGCGGCTGGATCACCTCCTTTCTAAGGAGCACCACGAAGAGCACTCCAATTGGTGGGGTGCGAGCCGTGAGGGGTCATCGTCTGTAGTGGACGAAGACCGGGTGCACGACAACAAGCAAAGCCAGACACACTATTGGGTCCTGAGGCAACACCCTCGGGTGCTGTCCCCCCATCTTGGTGGTGGGGTGTGGTGTTTGAGAATTGGATAGTGGTTGCGAGCATCAAAATGTATGCGTTGTCGTCCGCGGCAACGTGTTCTTTTTGTGCAATTTTATTCTTTGGTTTTTGTAGTGTTTGTAAGTGTCTAAGGGCGCATGGTGGATGCCTTGGCATCGAGAGCCGATGAAGGACG

>N027R

GATTGGGACGAAGTCGTAACAAGGTAGCCGTACCGGAAGGTGCGGCTGGATCACCTCCTTTCTAAGGAGCACCACGAAGAGCACTCCAATTGGTGGGGTGCGAGCCGTGAGGGGTCATCGTCTGTAGTGGACGAAGACCGGGTGCACGACAACAAGCAAAGCCAGACACACTATTGGGTCCTGAGGCAACACCCTCGGGTGCTGTCCCCCCATCTTGGTGGTGGGGTGTGGTGTTTGAGAATTGGATAGTGGTTGCGAGCATCAAAATGTATGCGTTGTCGTCCGCGGCAACGTGTTCTTTTTGTGCAATTTTATTCTTTGGTTTTTGTAGTGTTTGTAAGTGTCTAAGGGCGCATGGTGGATGCCTTGGCATCGAGAGCCGATGAAGGACGTGGGAGGCT

>N055R

GATTGGGACGAAGTCGTAACAAGGTAGCCGTACCGGAAGGTGCGGCTGGATCACCTCCTTTCTAAGGAGCACCACGAAAAGCACTCCAATTGGTGGGGTGCGAGCCGTGAGGGGTTCCCGTCTGTAGTGGACGGGGGCCGGGTGCACAACAGCAAATGATTGCCAGACACACTATTGGGCCCTGAGACAACACTCGGTCGATCCGTGTGGAGTCCCTCCATCTTGGTGGTGGGGTGTGGTGTTTGAGTATTGGATAGTGGTTGCGAGCATCTAGATGAGCGCATAGTCCTTAGGGCTGATGCGTTCGTCGAAATGTGTAATTTCTTCTTTGGTTTTTGTGTGTAAGTAAGTGTTTAAGGGCGCATGGTGGATGCCTTGGCATCGAGAGCCGATGAAGGACGTGGGAGGCT

>N062R

GATTGGGACGAAGTCGTAACAAGGTAGCCGTACCGGAAGGTGCGGCTGGATCACCTCCTTTTTAAGGAGCACCACGAAAAGCACTCCAATTGGTGGGGTGCGAGCCGTGAGGGGTTCCCGTCTGTAGTGGACGGGGGCCGGGTGCACAACAGCAAATGATTGCCAGACACACTATTGGGCCCTGAGACAACACTCGGTCGATCCGTGTGGAGTCCCTCCATCTTGGTGGTGGGGTGTGGTGTTTGAGTATTGGATAGTGGTTGCGAGCATCTAGATGAGCGCATAGTCCTTGTGGCTGATGCGCTCGTCGAAATGTGTAATTTCTTCTTTGGTTTTTGTGTGTAAGTAAGTGTTTAAGGGCGCATGGTGGATGCCTTGGCATCGAGAGCCGATGAAGGACGTGGGAGGCT

>N075R

GATTGGGACGAAGTCGTAACAAGGTAGCCGTACCGGAAGGTGCGGCTGGATCACCTCCTTTCTAAGGAGCACCACGAAAAGCACTCCAATTGGTGGGGTGCGAGCCGTGAGGGGTTCCCGTCTGTAGTGGACGGGGGCCGGGTGCACAACAGCAAATGATTGCCAGACACACTATTGGGCCCTGAGACAACACTCGGTCGATCCGTGTGGAGTCCCTCCATCTTGGTGGTGGGGTGTGGTGTTTGAGTATTGGATAGTGGTTGCGAGCATCTAGATGAGCGCATAGTCCTTGTGGCTGATGCGTTCGTCGAAATGTGTAATTTCTTCTTTGGTTTTTGTGTGTAAGTAAGTGTTTAAGGGCGCATGGTGGATGCCTTGGCATCGAGAGCCGATGAAGGACGTGGGAGGCT

>N089R

GATTGGGACGAAGTCGTAACAAGGTAGCCGTACCGGAAGGTGCGGCTGGATCACCTCCTTTCTAAGGAGCACCACGAAAAGCACTCCAATTGGTGGGGTGCGAGCCGTGAGGGGTTCCCGTCTGTAGTGGACGGGGGCCGGGTGCACAACAGCAAATGATTGCCAGACACACTATTGGGCCCTGAGACAACACTCGGTCGATCCGTGTGGAGTCCCTCCATCTTGGTGGTGGGGTGTGGTGTTTGAGTATTGGATAGTGGTTGCGAGCATCTAGATGAGCGCATAGTCCTTGTGGCTGATGCGCTCGTCGAAATGTGTAATTTCTTCTTTGGTTTTTGTGTGTAAGTAAGTGTTTAAGGGCGCATGGTGGATGCCTTGGCATCGAGAGCCGATGAAGGACGTGGGAGGCT

>N136T

GATTGGGACGAAGTCGTAACAAGGTAGCCGTACCGGAAGGTGCGGCTGGATCACCTCCTTTCTAAGGAGCACCACGAGAAACACTCCAATTGGTGGAGTGTGAGCCGTGAGGGGTTCTCGTCTGTAGAGGACGAGGGCCGGGTGCACAACAACAGGCAATCGCCAGACACACTATTGGGCCCTGAGACAACACTCGGCCGACTTTGGTCGACGTGGTGTCCCTCCATCTTGGTGGTGGGGTGTGGTGTTTGAGCATTGAATAGTGGTTGCGAGCATCTAGACGGATGCGACGCCCTCGGGCTGCGTGTTCGTCAAAAATGTGTAATTTTTCTTTTGGTTTTTGTGTTCGTAAGTGTTTAAGGGCACATGGTGGATGCCTTGGCATCGAGAGCCGATGAAGGACGTGGGAGGCT

>N162T

GATTGGGACGAAGTCGTAACAAGGTAGCCGTACCGGAAGGTGCGGCTGGATCACCTCCTTTCTAAGGAGCACCACGAGAAACACTCCAATTGGTGGAGTGTGAGCCGTGAGGGGTTCTCGTCTGTAGTGGACGAGGGCCGGGTGCACAACAACAGGCAATCGCCAGACACACTATTGGGCCCTGAGACAACACTCGGCCGACTTTGGTCGACGTGGTGTCCCTCCATCTTGGTGGTGGGGTGTGGTGTTTGAGCATTGAATAGTGGTTGCGAGCATCTAGACGGATGCGACGCCCTCGGGCTGCGTGTTCGTCAAAAATGTGTAATTTTTCTTTTGGTTTTTGTGTTCGTAAGTGTTTAAGGGCACATGGTGGATGCCTTGGCATCGAGAGCCGATGAAGGACGTGGGAGGCT

>N160T

GATTGGGACGAAGTCGTAACAAGGTAGCCGTACCGGAAGGTGCGGCTGGATCACCTCCTTTCTAAGGAGCACCACGAAAAGCGCTTCAATTGGTGAAGTGCGAGCCGTGAGGGGTTCTCGTCTGTAGTGGACGAAAACCGGGTGCACAACAGCAAATGATTGCCAGACACACTATTGGGCCCTGAGACAACACTCGGTCGATCCGTGTGGAGTCCCTCCATCTTGGTGGTGGGGTGTGGTGTTTGAGTATTGGATAGTGGTTGCGAGCATCTAGATGAGCGCGTGGTCCTTCGTGGCCGGCGTGTTCATCGAAATGTGTAATTTCTTTTTTAACTCTTGTGTGTAAGTAAGTGTTTAAGGGCGCATGGTGGATGCCTTGGCATCGAGAGCCGATGAAGGACGTGGGAGGCT

>N170T

GATTGGGACGAAGTCGTAACAAGGTAGCCGTACCGGAAGGTGCGGCTGGATCACCTCCTTTTTAAGGAGCACCACGAAAAGCACTTCAATTGGTGAAGTGCGAGCCGTGAGGGGTTCTCGTCTGTAGTGGGCGAAAACCGGGTGCACAACAGCAAATAATTGCCAGACACACTATTGGGCCCTGAGACAACACTCGGTCGAACCGTGTGGAGTCCCTCCATCTTGGTGGTGGGGTGTGGTGTTTGAGTATTGGATAGTGGTTGCGAGCATCTAGATGAACGCGTGGTCCTTCGTGGCCGTCGTGTTCATCGAAATGTGTAATTTCTTTTTTAACTCTTGTGTGTAAGTAAGTGTTTAAGGGCGCATGGTGGATGCCTTGGCATCGAGAGCCGATGAAGGACGTGGGAGGCT

>N218D

GATTGGGACGAAGTCGTAACAAGGTAGCCGTACCGGAAGGTGCGGCTGGATCACCTCCTTTCTAAGGAGCACCACGAGAAACACTCCAATTGGTGGAGTGTGAGCCGTGAGGGGTTCTCGTCTGTAGTGGACGAGGGCCGGGTGCACAACAACAGGCAATCGCCAGACACACTATTGGGCCCTGAGACAACACTCGGCCGACTTTGGTCGACGTGGTGTCCCTCCATCTTGGTGGTGGGGTGTGGTGTTTGAGCATTGAATAGTGGTTGCGAGCATCTAGACGGATGCGACGCCCTCGGGCTGCGTGTTCGTCAAAAATGTGTAATTTTTCTTTTGGTTTTTGTGTTCGTAAGTGTTTAAAGGCGCT

>N004R

AAATCAAACACCACACCCCCCCACCAAAGTGTGAAGGCACAACACGCCGACCCCCCCAACGGGATGCCACCCCCCGAAAGGGCACGGGCCTGTTGTCTCAAAGCCCAATAGTGTGTCTGGGAGCTTCTTCACGTTTGTTGTTGCACCAGACCCCCACCCA

>N005R

CAAATCAAACACCACACCCCACCACCAAAGTGAGGCAACAACACGCCGACACCCGCAACCGGATGCCACCCCCCAAAAAGGGGCACGGGCCTGTTGTCTCAAAGCCCAATAGTGTGTCTGGCAGTCAAAGAAGTTTGTTGTGCACCAAGCCGGCGCCACTACAGCGACCAGGCTCCTCACGGCTACACCCCCACCAATTGAGAGTGTTTATCGT

>N009R

TCCTTTCTAAGGAGCACCACGAAGAGCACTCCAATTGGTGGGGTGCGAGCCGTGAGGGGTCATCGTCTGTAGTGGACGAAGACCGGGTGCACGACAACAAGCAAAGCCAGACACACTATTGGGTCCTGAGGCAACACCCTCGGGTGCTGTCCCCCCATCTTGGTGGTGGGGTGTGGTGTTTGAGAATTGGATAGTGGTTGCGAGCATCAAAATGTATGCGTTGTCGTCCGCGGCAACGTGTTCTTTTTGTGCAATTTTATTCTTTGGTTTTTGTAGTGTTTGT

>N010R

GCGGCTGGATCACCTCCTTTCTATGGAGAATCGCTCTCGGCAACGAGAGCATTCAGATATGACTTCTTAAGAAGTCACCCTTAAAATCAGGTTTCGGCCTGTTACTCACTCGTTGGTCAGTTTTGAGAGTTCAACTCTCAAAACSCCTTTTCTTTTGGAGAGGCATTTGATCCTTGAAAACTAGATAACGAAACGAATTTGCGCAATTAGAAATATCCTTTTAGCTGAACTTGTGTCAGATCAAGTTTTTAAAAAAGTAGCGCGAAGGTTTTGGGATCTATCGATCCTTTGGAAGTTTGTTTCTACCTTTGACCGTTTAGTAGATCAAAGGGAAAC

>N014R

TGTGGGATCAGTTGCGGTTGGGAGATCAGTGCCAGCCCTGTAGTGGGCCGCTGGTGGGTGCACAACAAACGTTTGAAGTGGTGTGGGAACACTGCTTTGAGGAATCTGCCAACACACTATTGGGCTTTGAAGCAACAAGCCCGTTTGTTCCCTGGCCACTGTGTGTGGTGGGAGAGCGTGTTGTCGCCCTGTCTTTGGTGGTGGGGTGTGGTGTTTGATTTGTGGATAGTGGTTGCGAGC ATCT

>N015R

GGATCACTCCTTTCTAAAGAGCACCATTTTTTGTTCCCCCGCCCCCACACCCTTGTGGGATCAGTTGCGGTTGGGAGATCAGTGCCAGCACCTGTAGTGGGCCGCTGGTGGGTGCACAACAAACGTTAGAAGTGGTGTGGGAAACGCTGCTTTGAGGAACTAGCCAACACACTATTGGGCTTTGAGACAACAAGCCCGTTTGTTCCCTGGCCACTGTGTGTGGTGGGGAGCGTGTTGTCGCCCTGCTTTGGTGGTGGGGTGTGGTGTTTGATTTGTGGATAGTGGTTGCGAGCATCTCACAACAAATTGCTCACCTT

>N016R

ACAAAGCTCCCCCAAGGCACCCCTAAAAATTAAACACAACCTCAACTAAAGATATTACCTATTTAATCCCCCCGCCAGCGCAGGCAGCGTATCCATTGATGCTCGCAACCACTATCCAGTTCTCAAACACCACACCCCACCACCAAGGCGGTGGGACAACACCTGGAACAAGTCCGAGTGTTGCCTCAGGACCCAACAGTGTGTTGGTGGCCAACTTTGTTGTCATGCACCCGGCTCTCGCCCACTACAGACAAGAACCCCTCACGGCCTACGCCCCACCAGTTGGGGCGTTTTCGTGGTGCTCCTTAGAAAGGAGGTGATCCAGCCGCACCTTCCGGTACGGCTACCTTGTTACGACTTCGTCCCAATCTA

>N020R

CGGCTGGATCACCTCCTTTCTAAGGAGCACCACGAAAAGCACCCCAACTGGTGGGGTGCGAGCCGTGAGGGGTTCCCGTCTGTAGTGGACGGGGGCCGGGTGCGCAACAGCAAATGATTGCCAGACACACTATTGGGCCCTGAGACAACACTCGGTCCGTCCGTGTGGAGTCCCTCCATCTTGGTGGTGGGGTGTGGTGTTTGAGTATTGGATAGTGGTTGCGAGCATCTAGATGAGCGCATGGTCTTGGTGGCCGGCGTTCATCGAAATGTGTAATTTCTTTTTTAACTCTTGTGTGTAAGTAAGTGTTTAAGGGCGCATGGTGGATGCCTTGGCATCGAGAGCCGATGAAGGACGTGGGAGGCT

>N033R

AAGGTGCGGCTGGATCACCTCCTTTCTAAGGAGCACCACGAAAAGCACTCCAATTGGTGGGGTGCGAGCCTTGAGGGGTTCCCGTCTGTAGTGGACGGGGGCCGGGTGCACAACAGCAAATGATTGCCAGACACACTATTGGGCCCTGAGACAACACTCGGTCGATCCGTGTGGAGTCCCTCCATCTTGGTGGTGGGGTGTGGTGTTTGAGTATTGGATAGTGGTTGCGAGCATCTAGATGAGCGCATAGTCCTTGTGGCTGATGCGCTCGTCGAAATGTGTAATTTCTTCTTTGGTTTTTGTGTGTAAGTAAGTGTTTAAGGGCGCATGGTGGATGCCTTGGCATCGAGAGCCGATGAA GGACGTG

>N051R

AGGTGCGGCTGGATCACCTCCTTTCTAAGGAGCACCATTTTCCCCCGTCCCCGCACCATGTGGGAGTAGTGCGGTTGGGATATTTTTCGCCGGCGCCTGTAGTGGGTTGTCGGTGGTGCAGATTGTTTTTCCAACAACAACAGCTTTGATCATCAACCGGCACTGTCTCTTCGGGGGGGTGCTGGCCGGCTTATGCTGGGCACACTGTTGGGTCCTGAGGCAACAGGCCGGTTGTCGCCCTCTTTGGGGGTGGGTGTGTTGTCGCTCCATCTTGGTGGTGGGGTGTGGTGTTTGTTTTGTGGATAGTGGTTGCGAGCATCTAACAAGCGAGGCTTTGGTCTTGTTTGTTTTGCAATTTTTGTTTCTTGGTTTTTGTGATTTGTAAGTGTTTAAGGGCGCATGGTGGATGCCTTGGC

>N064R

GTGCGGCTGGATCACCTCCTTTCTAAGGAGCACCACGAAAAGCACTCCAATTGGTGGAGTGCGAGCCGTGAGGGGTTCTCGTCTGTAGTGGACGAAAGCCGGGTGCACAACAGCAAATGATTGCCAGACACACTATTGGGCCCTGAGACAACACTCGGTCGAACCGTGTGGAGTCCCTCCATCTTGGTGGTGGGGTGTGGTGTTTGAGTATTGGATAGTGGTTGCGAGCATCTAGATGAACGCGTGGCCCTTGTGGCCGGCGTGTTCATCGAAATGTGTATTTTCTTTTTTGGTTTTTGTGTGTAAGTAAGTGTT

>N069R

GGATCACCTCCTTTCTAAGGAGCACCACGAAAAGCACTCCAATTGGTGGGGTGCGAGCCGTGAGGGGTTCCCGTCTGTAGTGGACGGGGGCCGGGTGCACAACAGCAAATGATTGCCAGACACACTATTGGGCCCTGAGACAACACTCGGTCGATCCGTGTGGAGTCCCTCCATCTTGGTGGTGGGGTGTGGTGTTTGAGTATTGGATAGTGGTTGCGAGCATCTAGATGAGCGCATAGTCTTGTGGCTGATGCGCTCGTCGAAATGTGTAATTTCTTCTTTGGTTTTTGTGTGTAAGTAAGTGTTTAAGGGCGCATGGTGGATGCCTTGGCATCGAGAGCCGATGAAGGACGTGGGAGGC

>N097R

ACCTCCTTTCTAAGGAGCACCATTTTTTCCCCCGCGCCTCACATGGGTGAGGGTTCTCGTACCTCCTTTCTAAGGAGCACCATTTTTTCCCCCGCGCCTCACATGGGTGAGGGTTCTCGTACAGCAACAAGCGCGATCACCAACCGCCGGGAGCGTTATCGTTTCTGGTGGCCGGCCTTTGTGGGTTGGGCACACTGTTGGGTCCTGAGGCAACAGGCCTGTTGTTGCCCCCTTGGTGGGGGTGGGTGTGTTGTCGCTCCATCTTGGTGGTGGGGTGTGGTGTTTGTTTTGTGGATAGTGGTTGCGAGCATC

>N101R

CTGGTGGCCGGCTTTCGGGTTGGGCACACTGTTGGGTCCTGAGGCAACAGGCCCGGTTGTCGCCCCGTGGTGGGGTGGGGGTTGTTGTCGCTCCATCTTGGTGGTGGGGTGTGGTGTTTGTTTTGTGGATAGTGGTTGCGAGCATCTAACAAGCAAGATGTTTTGGTCTTGTTTGTTTTGCAATTTTTGTTTCTTGGTTTTTGTGTGTGTAAGTGTGTAAGGGCGCATGGTGGATGCCTTGGC

>N102R

CTCCTTTCTAGGAGCACCACGAAAAACACCTCCATCGGGGAGGTGTATGCCGTGAGGAACCCGTGTGCTGTAGTGGCGCCGGGTTGGGTGCACAACAAACGTGTCGGTGGCGGGGATAGCGCCGGCGAAAAACTTTGACCAGACACACTATTGGGCTTTGAGACAACAAGCCCG

>N104R

AGCACCGATTCGATTCCCCCGCCGTCCGCGAAGTCGTGGGCAGTAGTGCGGTTGGGATATTTCAGCCAGGCCCTGTAGTGGGTGTCTGGTGGGTGCAAATGACAAACGTTGAGCCGGCGCGGGAAAGCGCGGGTGATGGAACTGCCAGACACACTATTGGGCTTTGAGACAACAGGCCCGTGCCCCTTTTGTTGGGGGGGGTCTCGGTGTGGGGGTGGCCGTGTGGTTGCCCCTCTTTGTGGGGGGGGGGTGG

>N115R

CGGCTTGGTGCACAACAAACTTCTTTGACTGCCAGACACACTATTGGGCTTTGAGACAACAGGCCCGTGCCCCTTTTGGGGGGTGGCATCCGGTTGCGGGTGTCGGCGTGTTGTTGCCTCACTTTGGTGGTGGGGTGTGGTGTTTGATTTGTGGATAGTGGTTGCGAGCATCTAGCACGCATGGTGGTGGGCTGGGAGGCCTTGCGAGGTTTGCCGGTCTTGTTGTGTGTTGATGTGCAATTTCTTTTGAAACTCATTTTTGGTTTTTGTGTTGTAAGTGTTTAAGGGCGCATGGTGGATGCCTTGGCACTGGGAGCCGATGAAGGACGTGGGAGGCT

>N116R

ACCTCCTTTCTAAGGAGCACCATTTTTTCCCCCGCGCCTCACATGGGTGAGGGTTCTCGTGGTTGGGATACATTTGCCGGCGCCTGTAGTGGGTTGTCCGGTGGTGCAGATAATTTACGAACAGCAACAAGCGCGATCACCAACCGCCGGGAGCGTTATCGTTTCTGGTGGCCGGCCTTTGTGGGTTGGGCACACTGTTGGGTCCTGAGGCAACAGGCCTGTTGTTGCCCCCTTGGTGGGGGTGGGTGTGTTGTCGCTCCATCTTGGTGGTGGGGTGTGGTGTTTGTTTTGTGGATAGTGGTTGCGAGCATC

>N122T

CTCCTTTCTAAGGAGCACCACGAGAAACACTCCAATTGGTGGAGTGTGAGCCGTGAGGGGTTCTCGTCTGTAGTGGACGAGGGCCGGGTGCACAACAACAGGCAATCGCCAGACACACTATTGGGCCCTGAGACAACACTCGGCCGACTTTGGTCGACGTGGTGTCCCTCCATCTTGGTGGTGGGGTGTGGTGTTTGAGCATTGAATAGTGGTTGCGAGCATCTAGACGGATGCGACGCCCTCGGGCTGCGTGTTCGTCAAAAATGTGTAATTTTTCTTTTGGTTTTTGTGT

>N124T.

ACCTCCTTTCTAAGGAGCACCAATTTTTCCCCCGCGCCTCACACAGGTGAGGGTTCACGTGGTTGGGATACGGTTTGCCGGCGCCTGTAGTGGGTTGTCCGGTGGTGCAGATAATGTACAAACAGCAACAAGCTTGCGAAATCATCAACTGCCGGGAGTCTTCTCGACTTCTGGGGGCCGGCTTTCGTGTTGGGCACACTGTTGGGTCCTGAGGCAACAGGCCATGTGTCCCCCCTGTAGTGGTTGCGAGCATC

>N128T

CACCTCCTTTCTAAGGAGCACCACGAAAAGCGCTTCAATTGGTGAAGTGCGAGCCGTGAGGGGTTCTCGTCTGTAGTGGACGAAAACCGGGTGCACAACAGCAAATGATTGCCAGACACACTATTGGGCCCTGAGACAACACTCGGTCGATCCGTGTGGAGTCCCTCCATCTTGGTGGTGGGGTGTGGTGTTTGAGTATTGGATAGTGGTTGCGAGCATCTAGATGAACGCGTGGTCCTTCGTGGCCGGCGTGTTCATCGAAATGTGTAATTTCTTTTTTAACTCTTGTGTGTAAGTAAGTGTTTAAGGGCGCATGGTGGATGCCTTGGCATCGAGAGCCGATGAAGGACGTGGGAGGCT

>N129T

GGCTGGATCACCTCCTTTCTAAGGAGCACCACGAAAAGCACTCCAATTGGTGGGGTGCGAGCCGTGAGGGGTTCCCGTCTGTAGTGGACGGGGGCCGGGTGCACAACAGCAAATGATTGCCAGACACACTATTGGGCCCTGAGACAACACTCGGTCGATCCGTGTGGAGTCCCTCCATCTTGGTGGTGGGGTGTGGTGTTTGAGTATTGGATAGTGGTTGCGAGCATCTAGATGAGCGCATAGTCCTTGTGGCTGATGCGTTCGTCGAAATGTGTAATTTCTTCTTTGGTTTTTGTGTGTAAGTAAGTGTTTAAGGGCGCATGGTGGATGCCTTGGCATCGAGAGCCGATGAAGGACGTGGGAGGC

>N130T

CACCTCCTTTCTAAGGAGCACCACGAGAAACACTCCAATTGGTGGAGTGTGAGCCGTGAGGGGTTCTCGTCTGTAGTGGACGAGGGCCGGGTGCACAACAACAGGCAATCGCCAGACACACTATTGGGCCCTGAGACAACACTCGGCCGACTTTGGTCGACGTGGTGTCCCTCCATCTTGGTGGTGGGGTGTGGTGTTTGAGCATTGAATAGTGGTTGCGAGCATCTAGACGGATGCGACGCCCTCGGGCTGCGTGTTCGTCAAAAATGTGTAATTTTTCTTTTGGTTTTTGTGT

>N131T

TCACCTCCTTTCTAAGGAGCACCACGAGAAACACTCCAATTGGTGGAGTGTGAGCCGTGAACTATTGGGCCCTGAGACAACACTCGGCCGACTTTGGTCGACGTGGTGTCCCTCCATCTTGGTGGTGGGGTGTGGTGTTTGAGCATTGAATAGTGGTTGCGAGCATCTAGACGGATGCGACGCCCTCGGGCTGCGTGTTCGTCAAAAATGTGTAATTTTTCTTTTGGTTTTTGTGT

>N132T

CACCTCCTTTCTAAGGAGCACCACGAGAAACACTCCAATTGGTGGAGTGTGAGCCGTGAGGGGTTCTCGTCTGTAGTGGACGAGGGCCGGGTGCACAACAACAGGCAATCGCCAGACACACTATTGGGCCCTGAGACAACACTCGGCCGACTTTGGTCGACGTGGTGTCCCTCCATCTTGGTGGTGGGGTGTGGTGTTTGAGCATTGAATAGTGGTTGCGAGCATCTAGACGGATGCGACGCCCTCGGGCTGCGTGTTCGTCAAAAATGTGTAATTTTTCTTTTGGTTTTTGTGT

>N133T

GGCTGGATCACCTCCTTTCTAAGGAGCACCACGAGAAACACTCCAATTGGTGGAGTGTGAGCCGTGAGGGGTTCTCGTCTGTAGTGGACGAGGGCCGGGTGCACAACAACAGGCAATCGCCAGACACACTATTGGGCCCTGAGACAACACTCGGCCGACTTTGGTCGACGTGGTGTCCCTCCATCTTGGTGGTGGGGTGTGGTGTTTGAGCATTGAATAGTGGTTGCGAGCATCTAGACGGATGCGACGCCCTCGGGCTGCGTGTTCGTCAAAAATGTGTAATTTTTCTTTTGGTTTTTGTGT

>N141T

GCTGGATCACCTCCTTTCTAAGGAGCACCACGAAAAGCATCCCAATTGGTGGGATGCAGGCCGTGTGGAGTTCTCGTCTGTAGTGGACGAGGGCTGGGTGCACAACAACAAATAAGCCACACACACTATTGGGTCCTGAGACAACACTCGGGCGCTAGCACGAAGTGTTGTCCCTCCATCTTGGTGGTGGGGTGTGGTGTTTGAGAACTGGATAGTGGTTGCGAGCATCAACTGATCGCGTCGCCGTTCGCGGTGGCGTGTTCTTTTGTGCAATTTTAAATTCTTTGGTTTTTGTAGTGTTTGTAAGTGTCTAAGGGCGCATGGTGGATGCCTTGG

>N155T

GTGCGGCTGGATCACCTCCTTTCTAAGGAGCACCACGAAAAGCACTCCAATTGGTGGGGTGCGAGCCGTGAGGGGTTCCCGTCTGTAGTGGACGGGGGCCGGGTGCACAACAGCAAATGATTGCCAGACACACTATTGGGCCCTGAGACAACACTCGGTCGATCCGTGTGGAGTCCCTCCATCTTGGTGGTGGGGTGTGGTGTTTGAGTATTGGATAGTGGTTGCGAGCATCTAGATGAGCGCATAGTCCTTGTGGCTGATGCGTTCGTCGAAATGTGTAATTTCTTCTTTGGTTTTTGTGTGTAAGTAAGTGTTTAAGGGCGCATGGTGGATGCCTTGGCATCGAGAGCCGATGAAGGACGTGGGAGGC

>N158T

AGGTGCGGCTGGATCACCTCCTTTCTAAGGAGCACCACGAAAAGCACTCCAATTGGTGGGGTGCGAGCCGTGAGGGGTTCCCGTCTGTAGTGGACGGGGGCCGGGTGCACAACAGCAAATGATTGCCAGACACACTATTGGGCCCTGAGACAACACTCGGTCGATCCGTGTGGAGTCCCTCCATCTTGGTGGTGGGGTGTGGTGTTTGAGTATTGGATAGTGGTTGCGAGCATCTAGATGAGCGCATAGTCCTTAGGGCTGATGCGTTCGTCGAAATGTGTAATTTCTTCTTTGGTTTTTGTGTGTAAGTAAGTGTTTAAGGGCGCATGGTGGATGCCTTGGCATCGAGAGCCGATGAAGGACGT

>N161T

GTGCGGCTGGATCACCTCCTTTCTAAGGAGCACCACGAAAAGCACTTCAATTGGTGAAGTGCGAGCCGTGAGGGGTTCTCGTCTGTAGTGGGCGAAAACCGGGTGCACAACAGCAAATAATTGCCAGACACACTATTGGGCCCTGAGACAACACTCGGTCGAACCGTGTGGAGTCCCTCCATCTTGGTGGTGGGGTGTGGTGTTTGAGTATTGGATAGTGGTTGCGAGCATCTAGATGAACGCGTGGTCCTTCGTGGTCGTCGTGTTCATCGAAATGTGTAATTTCTTTTTTAACTCTTGTGTGTAAGTAAGTGTTTAAGGGCGCATGGTGGATGCCTTGGCATCGAGAGCCGATGAAGGACGTGGGAGGCT

>N163T

GTGCRGCTGGATCACCTCCTTTCTAAGGAGCACCACGAGAAACACTCCAATTGGTGGAGTGTGASCCKTGAGGGGTTCTCGTCTGTAGTGGACGAGGGCCGGGTGCACAACAACAGGCAATCGCCAGACACACTATTGGGCCCTGAGACAACACTCGGCCGACTTTGGTCGACGTGGTGTCCCTCCATCTTGGTGGTGGGGTGTGGTGTTTGAGCATTGAATAGTGGTTGCGAGCATCTAGACGGATGCGACGCCCTCGGGCTGCGTGTTCGTCAAAAATGTGTAATTTTTCTTTTGGTTTTTGTGT

>N167T

GAAGGTGCGGCTGGATCACCTCCTTTCTAAGGAGCACCACGAAAAGCACTCCAATTGGTGGAGTGCGAGCCGTGAGGGGTTCTCGTCTGTAGTGGACGAAAACCGGGTGCACAACAGCAAATGATTGCCAGACACACTATTGGGCCCTGAGACAGCACTCGGTCGATCCGTGTGGAGTCTCCCCATCTTGGTGGTGGGGTGTGGTGTTTGAGAAGTGGATAGTGGTTGCGAGCATCTAGATGAACGCGTGGTCCTTCGTGGCCGGCGTGTTCATCGAAATGTGTAATTTCTTCTTTGGTTTTTGTGTGTAAGTAAGTGTTTAAGGGCGCATGGTGGATGCCTTGGCATCGAGAGCCGATGAAGGACGTGGGAGGCT

>N172T

GTGCGGCTGGATCACCTCCTTTCTAAGGAGCACCACGAAAAGCACTCCAATTGGTGGGGTGCGAGCCGTGAGGGGTTCTCGTCTGTAGTGGACGGGACTCGGGTGCACAACAACAAGCAAGCCAGACACACTATTGGGTCCTGAGGCAACACTCGGGGTATCACCCGGCTGTTGTCCCACCATCTTGGTGGTGGGGTGTGGTGTTTGAGAATTGGATAGTGGTTGCGAGCATCAAACGAAGACGTCGCCGTTCACGGTGGCGTGTTCTTTTGTGCAATTTTATTCTTGGTTTTGTAGTGTTTGTAAGTGTCTAAGGGCGCATGGTGGATGCCTTGGCATCGAGAGCCGATGAAGGACGTGGGAGGC

>N197C

GGATCACCTCCTTTCTAAGGAGCACCACGAGAAACACTCCAATTGGTGGAGTGTGAGCCGTGAGGGGTTCTCGTCTGTAGTGGACGAGGGCCGGGTGCACAACAACAGGCAATCGCCAGACACACTATTGGGCCCTGAGACAACACTCGGCCGACTTTGGTCGACGTGGTGTCCCTCCATCTTGGTGGTGGGGTGTGGTGTTTGAGCATTGAATAGTGGTTGCGAGCATCTAGACGGATGCGACGCCCTCGGGCTGCGTGTTCGTCAAAAATGTGTAATTTTTCTTTTGGTTTTTGTGT

>N199C

GTTGTTGCCCTGCTTTGGTGGTGGGGTGTGGTGTTTGATTTGTGGATAGTGGTTGCGAGCATCTCGCAATGGATTGCCTCTCTTTGTGGGGGGTGGTTTGTTGTGTGATAATGCAATTTTATTCTTCCGAGAATATTTTTTGATCTGTTTTGTGTGTAAGTGTTTAAGGGCGCATGGTGGATGCCTTGGCACTGGGAGCCGATGAARGACGTGG

>N200C

CCTCCTTTCTAAGGAGCACCATTTTCCCCCGCGCCTCACACAGGTGAGGGTTCACGTGGTTGGGATACATTTCGCCGGCGCCTGTAGTGGGTTGTCCGGTGGTGCAGAAAATTTACGAGCAACAACAAACTTTCGAAATCATCAACCGCCAGGAGTTTTCGTGACTTCTGGTGGCCGGC

>N202C

CGGCTGGATCACCTCCTTTCTAAGGAGCACCACGAAAAGCACTCCAATTGGTGGGGTGCGAGCCGTGAGGGGTTCCTGCCTGTAGTGGGCGGGGCCGGGTGCGCAACAGCAAATGATTGCCACACACACTATTGGGCCCTGAAGCAACACTCGGATCGATTGAGTGGTTGTCCCCCCATCTTGGTGGTGGGGTGTGGTGTTTGAGAACTGGATAGTGGTTGCGAGCATCTAAATGAATGCGCTGCCGATGGTGGTGTGTTCGTTTTGTGTAATTTTTCTTTGGTTTTTGTGTTTGTAAGTGCTTAAGGGCGCATGGTGGATGCCTTGGCATCGAGAGCCGATGAAGGACGTGGGAGG

>N204C

ACCTCCTTTCTAAGGAGCACCACGAGAACCTGGCCCGCCCACATCGTGTGGGAGTTCGGTGACCTTGGTCGATTCGTTGGATGGCCTTCGCCTGTAGTGGGTGGGGGTCTGGTGCAACAACAAACATTCTTTATGACCGCCAGACACACTATTGGGCTTTGAGACA

>N205C

ACCTCCTTTCTAAGGAGCACCATTTTTTCCCCCGTCCCCGCAACCGTGCGGGAAGTGATGGTTGGGATAGGTTTGCCGGCGCCTGTAGTGGGTTGTCCGGTGGTGCAGATTGTTTACGAACAACAACAAGYTTTCAAAATCCTCCACTGGCGGGTGTTCTTGCGTCTCTGCGGGCTTTGTGTTGTGGGCACACTGGTGGGTCCTGAGGCAACAGGCCTGTTGTTGCCCCCTTGGTGGGGGTGGGTG

>N206C

TAGAACACGTCAGTCACAAGGACTACGCGTTCATCTAGATGCTCGCAACCACTATCCAATACTCAAACACCACACCCCACCACCAAGATGGAGGGACACCACACGGACTGACCGAGTGTTGTCTCAGGGCCCAATAGTGTGTCTAGCAATCATTTGCTGTTGTGCACCCGGCTCCCGTCCACTACAGACGAAAACCCCTCACGGCTCGCACCCCACCAATTGGRGTGCTTTTCGTGGTGCTCCTTAGAAAGGAGGTGATCCAGCCGCACCTTCCGGTACGGCTACCTTGTTACGAATC

>N210C

CAGACACACTATTGGGCTTTGAGACAACAAGCCCGCGGTGTCGTGCCTCGTTGGGGGTGTGGTGCCGGCCTTTCGTCCCCGGGTTGTTGGGGGTGGGGGGTTCTTGTTGTTGCCCTGCTTTGGTGGTGGGGTGTGGTGTTTGATTTGTGGATAGTGGTTGCGAGCATCT

>N211C

CAGACACACTATTGGGCTTTGAGACAACAAGCCCGCGGTGTCGTGCCTCGTTGGGGGTGTGGTGCCGGCCTTTCGTCCCCGGGTTGTTGGGGGTGGGGGGTTCTTGTTGTTGCCCTGCTTTGGTGGTGGGGTGTGGTGTTTGATTTGTGGATAGTGGTTGCGAGCATCT

>N219T

GGCTGGATCACCTCCTTTCTAAGGAGCACCACGAAAAGCACTCCAATTGGTGGGGTGCGAGCCGTGAGGGGTTCCCGTCTGTAGTGGACGGGGGCCGGGTGCACAACAGCAAATGATTGCCAGACACACTATTGGGCCCTGAGACAACACTCGGTCGATCCGTGTGGAGTCCCTCCATCTTGGTGGTGGGGTGTGGTGTTTGAGTATTGGATAGTGGTTGCGAGCATCTAGATGAGCGCATAGTCCTTGTGGCTGATGCGCTCGTCGAAATGTGTAATTTCTTCTTTGGTTTTTGTGTGTAAGTAAGTGTTTAAGGGCGCATGGTGGATGCCTTGGCATCGAGAGCCGATGAAGGACGT

>N224T

CGAGACCTGGGCCGGCCCCGCAGATCGCGGGATCAGCTGAGCTTTCAGGCGATTCGTTGGATGGCCTCGCACCTGTAGTGGGTGGGGGTCTGGTGCACTCAACAAACTTGGCGTGGGATGCGGGAAAGCATCTGCGGAAAATCATCAGACACACTATTGGGCTTTGAGACAACAGGCCCGTGCCCCTTTCGGGGGGTGGCTCCCGGTTGGTGGGGTCGGCGTGTTGTTGCCTCACTTTGGTGGTGGGGTGTGGTGTTTGATTTG

>N039R

CACCTCCTTTCTAAGGAGCACCACGAAAAGGGTGAGACACTGGGTCTTACCCGAGCCGTGAGGAACCGTGTGCGCTGTAGTGGCGCCGGCTTGGTGCACAAACTTGTAGAACTGCCAGACACACTATTGGGCTTTGAGACAACAGGCCCGCGCCCCTGTTGGGGGGGTGGCATCCGGTTGCGGGTGTCGGCGTGTTGTTGCTCCTCTTTGGTGGTGGGGTGTGGTGTTTGATTTGTGGATAGTGGTTGCGAGCATCTAGCACGCAGAATCGTGTGGTCTCACTCTTTGTGGGTGGGGCTGGTTTTGT

>N003R

GGCTGGATCACCTCCTTTCTAAGGAGCATTTAGTGCCGTTTCAAACCCGACTGTGGTTTGGCGGTCGCTCACATTATTGGGTGGAACACTGACACGGTAATTCTTATTGCCTGCTGGCCGGCTTGTCCGGTGGGTGGGGTTTCGTCGGCACACTGTTGGGTGTCTGAGAGGACACGTCCTTTCTGTTGCTGCTGCGCGTCAGCGCTCACGTACCACGGCCTTTCGGGGTGTGGGTGGGTGGCGGTGTGGTGGTGTGTGTTGTTTGAGAATTGTATAGTGGACGCGAGCATCAACCATGATGCCGGATCGTTTGTATCTGGTGGAGTGGTTTTTGTTTCTGTAATTTTACATGTTTCTGTGTAAGCATTTTGTTGTGTTTTTTTGGCGCTTGATGTCATGAAGGGCGCATCGGATTTGTGATGGCTTCGGTTGTTTCGGGTTTGGTGTGTGTTTTGTGTGTTGTAAGTGTCTAAGGGCGCACGGTGGATGCCTTGGCGCAAGGAGCCGATGAAGGACGTGGGAGGC

>N047R

AGGTGCGGCTGGATCACCTCCTTTCTAAGGAGCGTTCTCCAGTCGAGGGGCAACAGAGGTTGTCTTCTTGCTGGCAGAGGCCGTTTAGTCCCCAATCGTGGGACTGCGGTTGCTCATGGGTGGAACACTGACAAGTATTCTTCTTCACTTCTGCCGGCCCGAGTGCTGGTGGGAAGAGAGTATATCGGCATACTGTTGGGTCCTGAGAGAACACGTGAGTGATCTTCTCGAAGGAAGGAACGACAAGACCTGGGCGTGGTCATACCGCGGTGCGAGAGTGCCGGCTGGTGCCACATGAGTTCGGGTGTTGTGTGTTGTTTGAGAACTGCACAGTGGACGCGAGCATCTTTGTTGTAAGT

>N078R

CGGCTGGATCACCTCCTTTCTAAGGAGCGTTCTCCAGTCGAGGGGCAACAGAGGTTGTCTTCTTGCTGGCAGAGGCCGTTTAGTCCCCAATCGTGGGACTGCGGTTGCTCATGGGTGGAACACTGACAAGTATTCTTCTTCATCTGCTGGCGGCCCGAGTGCTGGTGGGAAGAGAGTTATATCGGCATACTGTTGGGTCCTGAGAGAACACGTGAGTGATCTTTCAAGGAAGAAACGACGAGCGGGAAACGCAGGTCATACCGCAGCGGGAACTTCGGTTCTGGTTGGTCCGGTGTCTGCACTGGTTTTCTGTTTGTGTGTTGTTTGAGAACTGCACAGTGGACGCGAGCATCTTTGTTGTAAGTGTTTAAGAGCGTACGGTGGATGCCTTGGCACCAGGAGCCGATGAAGGACGTAGGAGGCT

>N083R

AAGGAGCATCTCCCCACACACCCCGAAGAGTCGGGGAGCGTGTGAGGGCAGAGCCATTTCGGATTCACATGTAATCCGGTGGTGCTCATGGGTGGAACACTGACAAGTATTCTTCTTCACTACTGCCGGCCCGAGTGCTGGTGGGAAGAGAGTTATATCGGCATACTGTTGGGTCCTGAGAGAACACGTGAGTGATCTTTCAAGGAAGAAACGACGAGCGGGAAACGCAGGTCATACCGCAGCGGGAACTTCGGTTCTGGTTGGTCCGGTGTCTGCACGGGTTTTCTGTTTGTGTGTTGTTTGAGAACTGCACAGTG

>N222T

AGGTGCGGCTGGATCACCTCCTTTCTAAGGAGCATTCTCCACGCCGGCTCACAGAGGTGGGTGCGGGTTGTGGCAGAGCCATTACGAACTCGAATGTGGTTCGGTGGTTGCTCATGGGTGGAACGCTGACAAGCTCCATCACAGATGGCCGGTACTGTGTTTCGCGGTGCTGGTGGATATATCAACACACTGTTGGGTCCTGAAAGAACAGGCGTTCTTTCCAGGTAATAAACATGATCCGCTCGGATCTCCGTCATACCGCGGGAGCTTCTTTGAGGAGTTGCCGGTGGTGCGGGTGGTAGGTTCGGGTGGGTGTGTTGTTTGAGAACTGCACAGTGGACGCGAGCATCTTTGTTTTGTAAGTGTTTTAGAGCGTTCGGTGGATGCCTTGGCACCAGGAGCCGATGAAGGACGTGGGAGGCT

>N054R

ACAAAGCTCCCCCAAGGCACCCCTAAAAATTAAACACAACCTCAACTAAAGATATTACCTATTTAATCCCCCCGCCAGCGCAGGCAGCGTATCCATTGATGCTCGCAACCACTATCCAGTTCTCAAACACCACACCCCACCACCAAGGCGGTGGGACAACACCTGGAACAAGTCCGAGTGTTGCCTCAGGACCCAACAGTGTGTTGGTGGCCAACTTTGTTGTCATGCACCCGGCTCTCGCCCACTACAGACAAGAACCCCTCACGGCCTACGCCCCACCAGTTGGGGCGTTTTCGTGGTGCTCCTTAGAAAGGAGGTGATCCAGCCGCACCTTCCGGTACGGCTACCTTGTTACGACTTCGTCCCAATCTA

>N001R

ACAAAGCTCCCCCAAGGCACCCCTAAAAATTAAACACAACCTCAACTAAAGATATTACCTATTTAATCCCCCCGCCAGCGCAGGCAGCGTATCCATTGATGCTCGCAACCACTATCCAGTTCTCAAACACCACACCCCACCACCAAGGCGGTGGGACAACACCTGGAACAAGTCCGAGTGTTGCCTCAGGACCCAACAGTGTGTTGGTGGCCAACTTTGTTGTCATGCACCCGGCTCTCGCCCACTACAGACAAGAACCCCTCACGGCCTACGCCCCACCAGTTGGGGCGTTTTCGTGGTGCTCCTTAGAAAGGAGGTGATCCAGCCGCACCTTCCGGTACGGCTACCTTGTTACGACTTCGTCCCAATCTA

>N011R

TCCTTTCTAAGAGCACCACGAAAACGCCCCAACTGGTGGGGCGTAAGCCGTGAGGGGTTCTTGTCTGTAGTGGGCGAGAGCCGGGTGCATGACAACAAAGTTGGCCACCAACACACTGTTGGGTCCTGAGGCAACACTCGAGACTTGTTCCAGGTGTTGTCCCACCGCCTTGGTGGTGGGGTGTGGTGTTTGAGAACTGGATAGTGGTTGCGAGCATCAATGGATACGCTGCCGGCTAGCGGTGGCGTGTTCTTTGTGCAATATTCTTTGAGTTTTTGTTGTGTTTGTAAGTGTCTAAGGGCGCATGGTGGATGCCTTG

>N012R

GTGCGGCTGGATCACCTCCTTTCTAAGGAGCACCACGAAAACGCCCCAACTGGTGGGGCGTAGGCCGTGAGGGGTTCTTGTCTGTAGTGGGCGAGAGCCGGGTGCATGACAACAAAGTTGGCCACCAACACACTGTTGGGTCCTGAGGCAACACTCGGACTTGTTCCAGGTGTTGTCCCACCGCCTTGGTGGTGGGGTGTGGTGTTTGAGAACTGGATAGTGGTTGCGAGCATCAATGGATACGCTGCCGGCTAGCGGTGGCGTGTTCTTTGTGCAATATTCTTTGGTTTTTGTTGTGTTTGTAAGTGTCTAAGGGCGCATGGTGGATGCCTTGGCATCGAGAGCCGATGAAG

>N021R

CCTCCTTTCTAAGGAGCACCACGAAAACGCCCCAACTGGTGGGGCGTAGGCCGTGAGGGGTTCTTGTCTGTAGTGGGCGAGAGCCGGGTGCATGACAACAAAGTTGGCCACCAACACACTGTTGGGTCCTGAGGCAACACTCGGACTTGTTCCAGGTGTTGTCCCACCGCCTTGGTGGTGGGGTGTGGTGTTTGAGAACTGGATAGTGGTTGCGAGCATA

>N023R

GTGCGGCTGGATCACCTCCTTTCTAAGGAGCACCACGAAAAGCATCCCAATTGGTGGGGTGCGAGCCGTGAGGGGTTCTCGTCTGTATTGGACGAAAACCGGTTGCTCAACACCAAATGATTGCCAAACACACTATTGGGCCCTGAGACAACACTCGGTCTATCCGTGTGTATTCCCTCCATCTTGTTGGTGGGGTGTGTTGT

>N031R

GCGCCCTTAAACACTTACTTACACACAAAAACCAAAGAAGAAATTACACATTTCGACGAACACGCGACCGTTGCCGGCAACGCATCCGTCTAGATGCTCGCAACCACTATCCAATACTCAAACACCACACCCCACCACCAAGATGGAGGGACACCACACGGACTGGCCGAGTGTTGTCTCAGGGCCCAATAGTGTGTCTGGCAATCATTTGCTGTTGTGCACCCGGCCCCGCCCACTACAGGCGGGAACCCCTCACGGCTCGCACCCCACCAATTGGAGTGCTTTTCGTGGTGCTCCTTAGAAAGGAGGTGATCCAGCCGCACCTTCCGGTACGGCTACCTTGTTACGAC

>N038R

AAGGTGCGGCTGGATCACCTCCTTTCTAAGGAGCACCACGAAAACGCCCCAACTGGTGGGGCGTAGGCCGTGAGGGGTTCTTGTCTGTAGTGGGCGAGAGCCGGGTGCATGACAACAAAGTTGGCCACCAACACACTGTTGGGTCCTGAGGCAACACTCGGACTTGTTCCAGGTGTTGTCCCACCGCCTTGGTGGTGGGGTGTGGTGTTTGAGAACTGGATAGTGGTTGCGAGCATCAATGGATACGCTGCCGGCTAGCGGTGGCGTGTTCTTTGTGCAATATTCTTTGGTTTTTGTTGTGTTTGTAAGTGTCTAAGGGCGCATGGTGGATGCCTTGGCATCGAGAGCCGATGAAGGACGTGGG

>N041R

CCTCCTTTCTAAGGAGCACCACGAAAACGCCCCAACTGGTGGGGCGTAGGCCGTGAGGGGTTCTTGTCTGTAGTGGGCGAGAGCCGGGTGCATGACAACAAAGTTGGCCACCAACACACTGTTGGGTCCTGAGGCAACACTCGGACTTGTTCCAGGTGTTGTCCCACCGCCTTGGTGGTGGGGTGTGGTGTTTGAGAACTGGATAGTGGTTGCGAGCATCAATGGATACGCTGCCGGCTAGCGGTGGCGTGTTCTTTG

>N044R

CCTCCTTTCTAAGGGAGCACCACGAAAACGCCCCAACTGGTGGGGCGTAGGCCGTGAGGGGTTCTTGTCTGTAGTGGGCGAGAGCCGGGTGCATGACAACAAAGTTGGCCACCAACACACTGTTGGGTCCTGAGGCAACACTCGGACTTGTTCCAGGTGTTGTCCCACCGCCTTGGTGGTGGGGTGTGGTGTTTGAGAACTGGATAGTGGTTGCGAG

>N050R

AGGTGCGGCTGGATCACCTCCTTTCTAAGGAGCACCACGAAAACGCCCCAACTGGTGGGGCGTAGGCCGTGAGGGGTTCTTGTCTGTAGTGGGCGAGAGCCGGGTGCATGACAACAAAGTTGGCCACCAACACACTGTTGGGTCCTGAGGCAACACTCGGACTTGTTCCAGGTGTTGTCCCACCGCCTTGGTGGTGGGGTGTGGTGTTTGAGAACTGGATAGTGGTTGCGAGCATCAATGGATACGCTGCCGGCTAGCGGTGGCGTGTTCTTTGTGCAATATTCTTTGGTTTTTGTTGTGTTTGTAAGTGTCTAAGGGCGCATGGTGGATGCCTTGGCATCGAGAGCCGATGAAGGACGTGGGAGG

>N052R

CCGGAAAGTGCGGCTGGACCCTCCTTTCTAAGGAGCACCACTCTACGCCCCAACTGGTGGGGCGTAGACCTCGTGGGATTCTTGTCTGTAGTGGGCGATAGCCCGGTGAATGACAACAAGTTGGCCACCAACACATTGTTGGGTCCTGACCTTCACTTTGACTTGTTCCAGGTGTTGTCCCACCCCCTTGGTGGTGGGGTGTGGTGTCAATTAAATATATTCCTTTTAGCTGAACTTATGTCAACACTGCCGGCTAACAGTGGCATGTTCTTTGTGCAATAATCTTTGATTTCAGTTACTTTTGAAATTGTCTTACGGCCCATGGTGCATTCATGGATCGAGAGCCTATGAACGAC

>N065R

CCCTTAAACACTTACTTACACACAAAAACCAAAGAAGAAATTACACATTTCGACGAGCGCATCAGCCACAAGGACTATGCGCTCATCTAGATGCTCGCAACCACTATCCAATACTCAAACACCACACCCCACCACCAAGATGGAGGGACTCCACACGGATCGACCGAGTGTTGTCTCAGGGCCCAATAGTGTGTCTGGCAATCATTTGCTGTTGTGCACCCGGCCCCCGTCCACTACAGACGGGAACCCCTCACGGCTCGCACCCCACCAATTGGAGTGCTTTTCGTGGTGCTCCTTAGAAAGGAGGTGATCCAGCCGCACCTTCCGGTACGGCTACCTTGTTACGACTTCGTCCCAATC

>N066R

CCTCCTTTCTAAGGAGCACCACGAAAACGCCTCAACTGGTGGGGCGGAGGCCGTGAGGGGTTCTGGTCTGTAGGGGACGAGAGCCGGGTGCATGACAACAAAGTGGGCCACCAACACACTGTTGGGTCCTGAGGCAACACTCGGACTTGTTCCGGGTGTTCCCTCCCTCCCTTGGTGGTGGGGTGTGGTGTTAGAAAAGTGTATAGTGTGTGAGAATCTCAATGAATACGCTGCCCTTTAGCGGTGGCGTGTTCTTTGTGCAATATTCTTTGTTTTTTGTTGTGTTTGTAAGTGTCTAAGGGCGCGTGGTGGATGCCTTGGCWTCGAGAGCCGATGAAAGAAATGAGAGGCT

>N068R

CGGCTGGATCACCTCCTTTCTAAGGAGCACCACGAAAACGCCCCAACTGGTGGGGCGTAGGCCGTGAGGGGTTCTTGTCTGTAGTGGGCGAGAGCCGGGTGCATGACAACAAAGTTGGCACCAACACACTGTTGGGTCCTGAGGCAACACTCGGACTTGTTCCGGGTGTTGTCCCACCGCCTTGGTGGTGGGGTGTGGTGTTTGAGAACTGGATAGTGGTTGCGAGCATCAATGAATACGCTGCCCGTTAGGGGTGGCGTGTTCTTTGTGCAATATTCTTTGGTTTTTGTTGTGTTTGTAAATGTCTAAGGGCGCATGGTGGATGCCTTGGCATCGAGAGCCGATGAAGGACATGGGAGG

>N070R

GGCTGGATCACCTCCTTTCTAAGGAGCACCACGAAAACGCCCCAACTGGTGGGGCGTAGGCCGTGAGGGGTTCTTGTCTGTAGTGGGCGAGAGCCGGGTGCATGACAACAAAGTTGGCCACCAACACACTGTTGGGTCCTGAGGCAACACTCGGACTTGTTCCAGGTGTTGTCCCACCGCCTTGGTGGTGGGGTGTGGTGTTTGAGAACTGGATAGTGGTTGCGAGCATCAATGGATACGCTGCCGGCTAGCGGTGGCGTGTTCTTTGTGCAATATTCTTTGGTTTTTGTTGTGTTTGTAAGTGTCTAAGGGCGCATGGTGGATGCCTTGGCATCGAGAGCCGATGAAGGACGTGGGAGGC

>N074R

CGGAAGGTGCGGCTGGATCACCTCCTTTCTAAGGAGCACCACGAAAAGCACTCCAATTGGTGGGGTGCGAGCCGTGAGGGGTTCCCGTCTGTAGTGGACGGGGGCCGGGTGCACAACAGCAAATGATTGCCAGACACACTATTGGGCCCTGAGACAACACTCGGTCGATCCGTGTGGAGTCCCTCCATCTTGGTGGTGGGGTGTGGTGTTTGAGTATTGGATAGTGGTTGCGAGCATCTAGATGAGCGCATAGTCCTTGTGGCTGATGCGTTCGTCGAAATGTGTAATTTCTTCTTTGGTTTTTGTGTGTAAGTAAGTGTTTAAGGGCGCATGGTGGATGCCTTGGCATCGAGAGCCGATGAAGGACGTGGGAGGCT

>N076R

CACAAAAACCAAAAATGAGTTTCAAAAGAAATTGCACATCAACACACAACAAGACCGGAAACCCGAAGGCCCCCAGCCACACTGCGTGCTAGATGCTCGCAACCACTATCCACAAATCAAACACCACACCCCACCACCAAAGTGAGGCAACAACACGCCGACACCCGCAACCGGATGCCACCCCCCAAAAAAGGGGCACGGGCCTGTTGTCTCAAAGCCCAATAGTGTGTCTGGCAGTCCCATCACCGCAAAATCCCTTGCGGCTCAACGTTTGTTGTGCACCAAACCGTGCCCACTACAGGCAACCGGTTCCTCACGGCTCGGGTAAGACCCAGTGTCTCAACCCTTTTCGTGGTGCTCCTT

>N077R

AGCCACAAGGACTATGCGCTCATCTAGATGCTCGCAACCACTATCCAATACTCAAACACCACACCCCACCACCAAGATGGAGGGACTCCACACGGATCGACCGAGTGTTGTCTCAGGGCCCAATAGTGTGTCTGGCAATCATTTGCTGTTGTGCACCCGGCCCCCGTCCACTACAGACGGGAACCCCTCACGGCTCGCACCCCACCAATTGGAGTGCTTTTCGTGGTGCTCCTTAGAAAGGAGGTGATCCAGCCGCACCTTCCGGTACGGCTACCTTGTTACGACTTCGTCCCAATC

>N085R

CTCCTTTCTAAGGAGCACCACGAAAAGCACTCCAATTGGTGGGGTGCGAGCCGTGAGGGGTTCCCGTCTGTAGTGGACGGGGGCCGGGTGCACAACAGCAAATGATTGCCAGACACACTATTGGGCCCTGAGACAACACTCGGTCGATCCGTGTGGAGTCCCTCCATCTTGGTGGTGGGGTGTGGTGTTTGAGTATTGGATAGTGGTTGCGAGCATCTAGATGAGCGCATAGTCCTTGTGGCTGATGCGCTCGTCAAAA

>N096R

CCGTGAGGAACCGGTTGCCTGTAGTGGGCACGGTTTGGTGCACAACAAACGTTGAGCCGCAAGGGATTTTGCGGTGATGGGACTGCCACACACACTATTGGGCTTTGACACAACAGGCCCGTGCCCCTTTTTTGGGGGGTGGCATCCGGTTGCGG

>N137T

ACACAAAAACCAAAAGAAAAATTACACATTTTTGACGAACACGCAGCCCGAGGGCGTCGCATCCGTCTAGATGCTCGCAACCACTATTCAATGCTCAAACACCACACCCCACCACCAAGATGGAGGGACACCACGTCGACCAAAGTCGGCCGAGTGTTGTCTCAGGGCCCAATAGTGTGTCTGGCGATTGCCTGTTGTTGTGCACCCGGCCCTCGTCCACTACAGACGAGAACCCCTCACGGCTCACACTCCACCAATTGGAGTGTTTCTCGTGGTGCTCCTTAGAAAGGAGGTGATCCAGCCGCACCTTCCGGTACGGCTACCTTGTTACGAC

>N143T

GGCTGGATCACCTCCTTTCTAAGGAGCACCACGAAAACGCCCCAACTGGTGGGGCGTAGGCCGTGAGGGGTTCTTGTCTGTAGTGGGCGAGAGCCGGGTGCATGACAACAAAGTTGGCCACCAACACACTGTTGGGTCCTGAGGCAACACTCGGACTTGTTCCAGGTGTTGTCCCACCGCCTTGGTGGTGGGGTGTGGTGTTTGAGAACTGGATAGTGGTTGCGAGCATCAATGGATACGCTGCCGGCTAGCGGTGGCGTGTTCTTTGTGCAATATTCTTTGGTTTTTGTTGTGTTTGTAAGTGTCTAAGGGCGCATGGTGGATGCCTTGGCATCGAGAGCCGATGAAGGACGTGGGAGG

>N178D

TTACTTACACACAAAAACCAAAGAAGAAATTACACATTTCGATGAACACGTCGACCGCGAAGGACCACGCGTTCATCTAGATGCTCGCAACCACTATCCAATACTCAAACACCACACCCCACCACCAAGATGGAGGGACTCCACACGGATCGACCGAGTGTTGTCTCAGGGCCCAATAGTGTGTCTGGCAATCATTTGTTGTTGTGCACCCGGTTTTCGTCCACTACAGACGAGAACCCCTCACGGCTCGCACT

>N187D

ATCACCTCCTTTCTAAGGAGCACCACGAAAACGCCCCAACTGGTGGGGCGTAGGCCGTGAGGGGTTCTTGTCTGTAGTGGGCGAGAGCCGGGTGCATGACAACAAAGTTGGCCACCAACACACTGTTGGGTCCTGAGGCAACACTCGGACTTGTTCCAGGTGTTGTCCCACCGCCTTGGTGGTGGGGTGTGGTGTTTGAGAACTGGATAGTGGTTGCGAGCATCAATGGATACGCTGCCGGCTAGCGGTGGCGTGTTCTTTGTGCAATATTCTTTGGTTTTTGTTGTGTTTGTAAGTGTCTAAGGGCGCATGGTGGATGCCTTGGCATCGAGAGCCGATGAAGGACGTGGGAGGCT

>N201C

CCTCCTTTCTAAGGAGCACCATTTTCCCCCGCGCCTCACACAGGTGAGGGTTCACGTGGTTGGGATACATTTCGCCGGCGCCTGTAGTGGGTTGTCCGGTGGTGCAGAAAATTTACGAGCAACAACAAACTTTCGAAATCATCAACCGCCAGGAGTTTTCGTGACTTCTGGTGGCCGGC

>N225T

CGAGACCTGGGCCGGCCCCGCAGATCGCGGGATCAGCTGAGCTTTCAGGCGATTCGTTGGATGGCCTCGCACCTGTAGTGGGTGGGGGTCTGGTGCACTCAACAAACTTGGCGTGGGATGCGGGAAAGCATCTGCGGAAAATCATCAGACACACTATTGGGCTTTGAGACAACAGGCCCGTGCCCCTTTCGGGGGGTGGCTCCCGGTTGGTGGGGTCGGCGTGTTGTTGCCTCACTTTGGTGGTGGGGTGTGGTGTTTGATTTG
